# Effects of Ion-Releasing Tooth-Coating Material on Demineralization of Bovine Tooth Enamel

**DOI:** 10.1155/2014/463149

**Published:** 2014-01-21

**Authors:** Koji Kawasaki, Masaki Kambara

**Affiliations:** Preventive and Community Dentistry, Osaka Dental University, 8-1 Kuzuha Hanazono, Hirakata, Osaka 573-1121, Japan

## Abstract

We compared the effect of a novel ion-releasing tooth-coating material that contained S-PRG (surface-reaction type prereacted glass-ionomer) filler to that of non-S-PRG filler and nail varnish on the demineralization of bovine enamel subsurface lesions. The demineralization process of bovine enamel was examined using quantitative light-induced fluorescence (QLF) and electron probe microanalyzer (EPMA) measurement. Ion concentrations in demineralizing solution were measured using inductively coupled plasma atomic (ICP) emission spectrometry and an ion electrode. The nail varnish group and the non-S-PRG filler group showed linear demineralization. Although the nail varnish group and the non-S-PRG filler group showed linear demineralization, the S-PRG filler group did not. Further, plane-scanning by EPMA analysis in the S-PRG filler group showed no changes in Ca ion distribution, and F ions showed peak levels on the surface of enamel specimens. Most ions in the demineralizing solution were present at higher concentrations in the S-PRG filler group than in the other two groups. In conclusion, only the S-PRG filler-containing tooth-coating material released ions and inhibited demineralization around the coating.

## 1. Introduction

In recent years, oral health in developed countries has improved, with the majority of people keeping more sound teeth for longer duration [1–7]. In particular, considerable interest has been directed at detecting caries at early stages, with the development of the International Caries Detection and Assessment System (ICDAS) [8, 9] and quantitative light-induced fluorescence (QLF) method [10, 11]. In current daily dental practice and oral health care programs, the inhibition of initial tooth enamel demineralization and the promotion of remineralization are the most important targets [12–14].

Surface-reaction type prereacted glass-ionomer (S-PRG) filler [15, 16] has been reported to have biological efficacy in reducing dental plaque formation [17, 18], inhibition of dentin demineralization [19], fluoride release and recharge potential [20], and prevention of demineralization in surrounding orthodontic brackets [21]. These efficacies might be due to the ability of S-PRG filler to release various ion species (fluoride, strontium, aluminum, sodium, etc.) as well as its capacity as an acid buffer [22]. S-PRG filler can therefore be found in various dental products, such as composite resin, root canal sealer, orthodontic resin bonding systems, and denture base resin [19, 23–28].

We developed a novel ion-releasing tooth-coating material containing S-PRG filler, which was developed for surface-coating sound teeth and which consists of a base liquid containing S-PRG filler and an active liquid containing carboxylic and phosphonic adhesive monomers. In clinical practice, the dentist or dental hygienist would mix these two liquids, apply the mixture to the tooth surface with a brush, and polymerize it via light irradiation. To our knowledge, the inhibitory effect of an ion-releasing tooth-coating material that contains S-PRG on the demineralization of tooth enamel has not been reported.

Here, in order to clear this point, we investigated various coating material applications on bovine tooth enamel using the quantitative light-induced fluorescence (QLF) method [29–31] to longitudinally monitor the demineralization process and an electron probe microanalyzer (EPMA) [32] for mineral mapping of demineralized enamel.

## 2. Materials and Methods

### 2.1. Preparation of Enamel Specimens

Extracted bovine incisor teeth were used as a source of enamel. Using a core drill to excise bovine enamel specimens (5 mm in diameter), we obtained specimens with an enamel layer thickness of approximately 1.0 mm and a dentine layer thickness of approximately 2.0 mm. Enamel surfaces were abraded using carbide paper of 600 to 1,500 grit and were then polished with gamma alumina polishing paste (grain diameter, 0.05 *μ*m).

### 2.2. Tooth-Coating Materials and Demineralization of Enamel Specimens

Ten samples from each enamel specimen group were painted with an acid-resistant nonfluorescent varnish (control group), S-PRG filler-containing tooth-coating material (PRG Barrier Coat; Shofu Inc., Kyoto, Japan), or non-S-PRG filler-containing tooth-coating material (Shofu Inc.), leaving an enamel window of approximately 2 × 2 mm^2^ (coated area was approximately 15 mm^2^ in size and approximately 10 *μ*m in thickness [33]). In separate bottles, specimens were then exposed to 14 mL of a demineralization solution for 96 hours at 37.0°C. The demineralization solution contained 0.1 M lactic acid (Kishida Chemical, Osaka, Japan) and 0.2 g/L carboxymethyl cellulose sodium salt (Kishida Chemical), was 50% saturated with hydroxyapatite (Nihon Chemical, Tokyo, Japan), and was adjusted to a pH of 5.0 [34].

### 2.3. QLF Measurement

Images of specimens containing white spot lesions were acquired with a QLF-Clin system (Inspektor Research Systems BV, Amsterdam, The Netherlands) equipped with QLF.exe evaluation software (version 2.00h) to digitize and quantify the images. Digital images were obtained every 24 h over a 96 h period. Specimens were illuminated with violet-blue light (*λ* = 390–430 nm). A CCD camera with a yellow high-pass filter (*λ* = 520 nm) was fixed with a stand in order to provide optimal illumination of the specimen surface. Quantitative results were obtained for the following parameters: mean fluorescence loss over the lesion (%), area of the lesion (mm^2^), and total fluorescence loss over the lesion (Δ*Q* in %·mm^2^). These parameters were determined using a threshold of 5% fluorescence radiance loss [35]. Δ*Q* is comparable to the total mineral loss from lesions, as measured via longitudinal microradiography [36]. The three different coating materials remained on the tooth enamel surface throughout the 96 h test period. All analyses of digital images were conducted in the 2 × 2 mm^2^ area in the center of the enamel specimens.

### 2.4. Ion Release from Tooth-Coating Material

After 96 h, demineralized solution was subjected to analysis of ion concentration (B, Al, Ca, P, Si, and Sr) using an ICP emission spectrometer (ICPS-8000; Shimadzu, Kyoto, Japan). Analysis of pH and fluoride ion concentration was performed with an ion electrode (pH: Model 9102BNWP and F: Model 9609BN; Orion Research Inc., Boston, MA, USA). TISAB III (Orion Research) was added to the solution in order to obtain a constant background ionic strength for fluoride.

### 2.5. Chemical Composition Analysis of Demineralized Enamel

Demineralized enamel specimens were vertically sectioned with a low-speed diamond cutter and mounted on aluminum stubs. Sectioned specimens were sputter-coated with a 300 Å gold layer using an ion coater (IC-50; Shimadzu) and analyzed using a wavelength-dispersive X-ray spectroscopy electron probe microanalyzer with an image observation function (SEM-EPMA, EPMA1601; Shimadzu). For morphological observation, the subsurface lesions of enamel specimens were analyzed under SEM-EPMA at an accelerating voltage of 15 kV. Chemical component bulk analysis and element mapping were carried out using SEM-EPMA for the subsurface area (60–70 *μ*m away from the interface). Distributions of Ca and F in the enamel were measured using the element line scan from the interface to the direction of inner enamel with an approximate range of 100 *μ*m.

### 2.6. Statistics

Statistical calculations, analysis of variance (ANOVA) followed by Tukey's all-pairwise-comparison test, were performed with software package SPSS version 11.0J for Windows XP (SPSS Inc., Chicago, IL, USA).

## 3. Results

**Figure 1:**
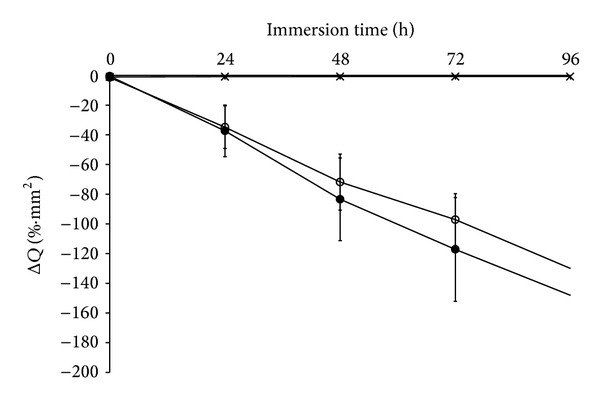
Mean Δ*Q* for the 3 groups immersed in demineralized solution for 96 h. ×: control group, ●: non-S-PRG filler group, and ○: S-PRG filler group.

**Figure 2:**
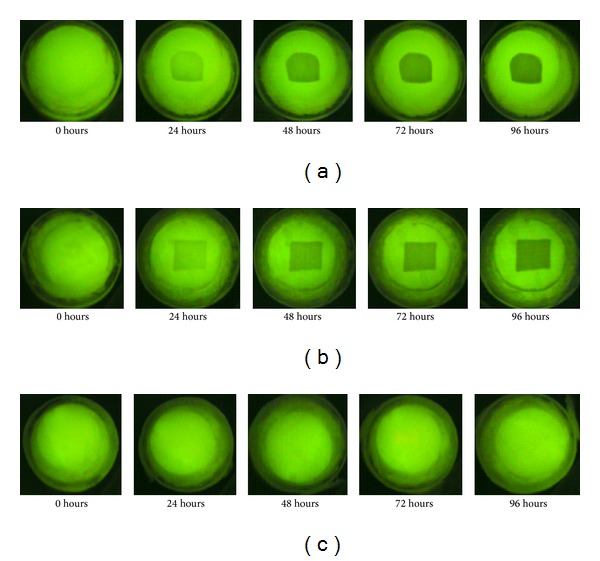
Digital images of QLF after the 96 h demineralizing process. (a) Control group, (b) non-S-PRG filler group, and (c) S-PRG filler group.

The mean Δ*Q* for the three groups (control group, non-S-PRG filler group, and S-PRG filler group), immersed in demineralized solution for 96 hours, is shown in Figure 1. QLF digital images are shown in Figure 2. Mean Δ*Q* at 96 h was −148.1 ± 38.9%·mm^2^ for the control group, −130.0 ± 21.2%*·*mm^2^ for the non-S-PRG filler group, and −0.8 ± 0.4%·mm^2^ for the S-PRG filler group. The differences between the S-PRG filler and the other two groups were statistically significant (*P* < 0.001). In the S-PRG group, no signs of demineralization were observed in QLF digital images (Figure 2).

The pH values of the demineralized solution did not change after 96 h of immersion. Mean ion concentrations in the demineralized solution, as assessed using ICP emission spectrometry, are shown in Table 1. While no significant differences were noted between the control group and the non-S-PRG filler group, five ions (Al, B, Si, Sr, and F) were present at higher concentrations in the S-PRG filler group than in the other two groups, with the concentration of F ion being particularly elevated. According to the QLF image, the Ca and P of tooth enamel were dissolved in the demineralization solution in both the control and non-S-PRG filler groups. Concentrations of Ca and P in solution may have therefore increased. However, the concentration of the Ca and P in the demineralizing solution in both the control and non-S-PRG filler groups showed the approximate value between S-PRG filler group (Table 1). The influence of dissolving tooth enamel appears to be limited.

**Figure 3:**
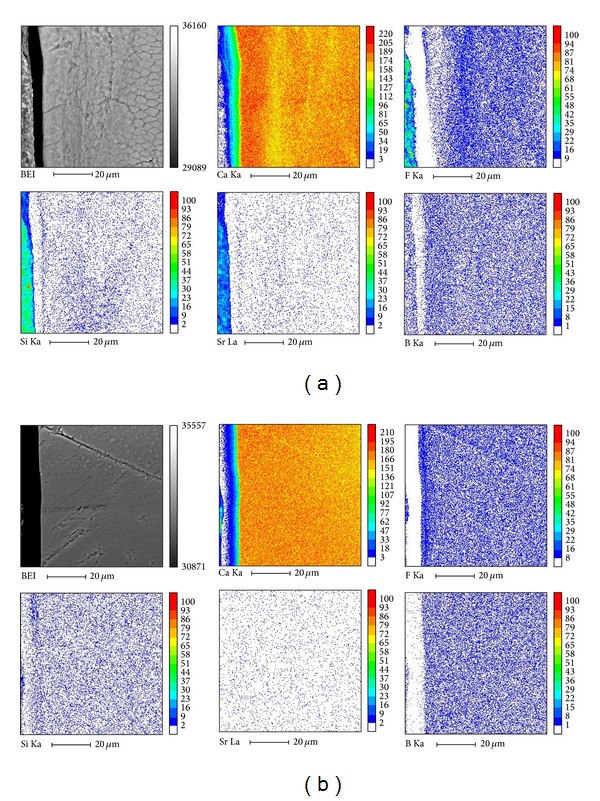
Plane-scanning analysis via EPMA. Upper: control; lower: S-PRG filler-containing tooth-coating material. Upper left: SEM image; upper center: calcium; upper right: fluoride; lower left: silica; lower center: strontium; lower right: boron.

**Figure 4:**
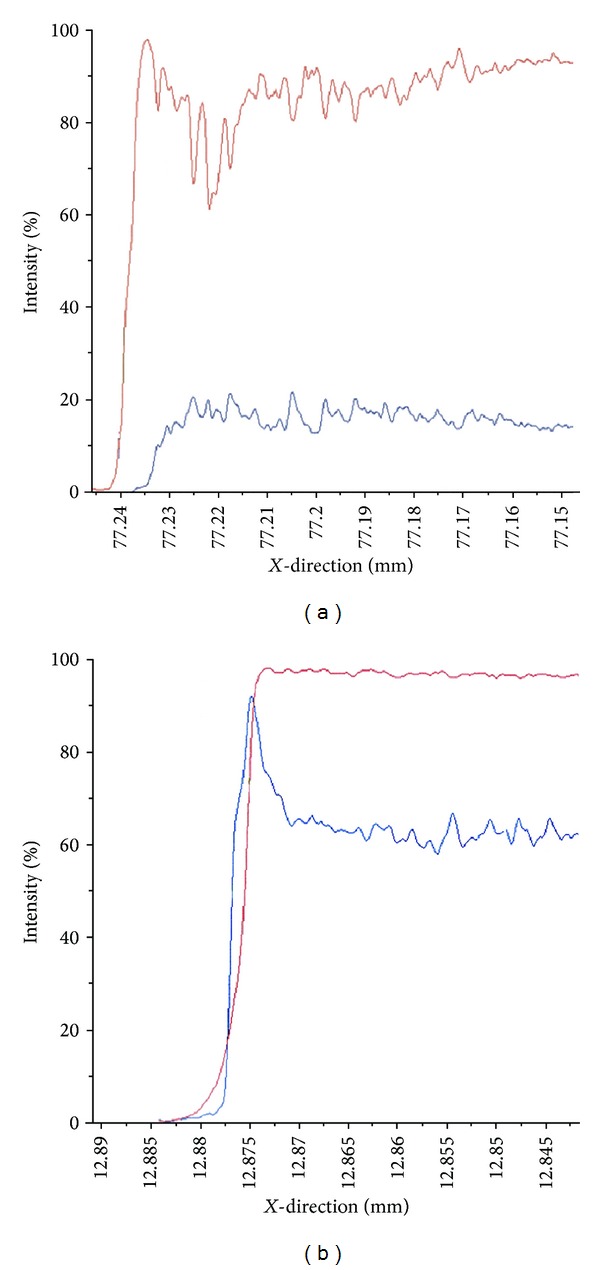
Line scanning analysis by EPMA. Upper: control; lower: S-PRG filler-containing tooth-coating material; red line: Ca; blue line: F.

Plane-scanning analysis via EPMA is shown in Figures 3(a) and 3(b). As the images for the control and non-S-PRG filler group were nearly identical, only the control and S-PRG filler groups are shown. In both groups, a scarcity of B, Si, and Sr ions was observed in enamel specimens. Results of line-scanning analysis for Ca and F are shown in Figures 4(a) and 4(b). In the control group, concentrations of Ca ions were lower on the subsurface of enamel specimens than sound enamel, and F ion concentrations were relatively low as well. In the S-PRG group, however, no marked changes were noted in Ca ion distribution, and F ions peaked on the surface of enamel specimens.

## 4. Discussion

Mukai et al. [27] reported that an all-in-one adhesive system containing S-PRG filler was able to form protective layers and that it protected dentin against further demineralization in the case of secondary marginal dental caries. Kamijo et al. [20] similarly reported that denture base resins containing S-PRG filler have relatively good fluoride recharge and release capacities, which assist in preventing caries. Most studies have investigated the preventative effects of dentin demineralization after restorative treatment. In our *in vitro* study, we investigated the inhibitory effects of a tooth-coating material that contained S-PRG filler on the demineralization of bovine tooth enamel. It should be noted that bovine tooth enamel is demineralized more rapidly than human tooth enamel [37]. However, many *in vitro* studies use bovine tooth [38–40], and it is well known that the human enamel has individual difference. We did not use human tooth enamel specimens given the difficulty in acquiring such specimens.

Based on QLF measurement, tooth-coating material containing S-PRG filler protected the enamel surface against demineralization. In a previous study [41], APF-gel-treated enamel specimens also showed almost no demineralization. The S-PRG filler-containing coating material blocked demineralization on the coated areas and inhibited demineralization of the surrounding areas. Featherstone et al. [42] reported that the continual presence of low concentrations of fluoride (0.1–0.5 ppm) in whole saliva is critical in the inhibition of demineralization and promotion of remineralization. In the present study, the fluoride concentration in demineralized solution was 0.34 ppm, suggesting that the tooth-coating material containing S-PRG filler might release ions and inhibit enamel demineralization.

Table 1 shows the mean ion concentrations in the demineralizing solution. Al, B, Si, Sr, and F ion concentrations in the demineralized solution of S-PRG filler group were higher than in the other two groups. While pH values of the demineralizing solution did not change after 96 h of immersion, the elevated ion concentrations indicate that the demineralizing solution of S-PRG filler group may be induced to behave differently during the demineralization process. Of note, our *in vitro* study employed a static model to induce demineralization, which may have resulted in an overestimation of the effect of the coating due to a build-up of released ions in the demineralizing solution. The results of this study might not reflect the *in vivo* conditions in which there is constant salivary clearance.

We also investigated the influence of ions released from S-PRG filler using EPMA as the gold coating was anticipated to have an effect on quantitative analysis via scattering of electrons due to its high atomic number and density. Line-scanning analysis showed that fluoride saturated the sound teeth surface in S-PRG filler-treated specimens. High-dosage APF-gel application on the enamel surface produces a fluoride calcium layer on the tooth surface. Rølla and Øgaard [43] reported that reductions in pH from 7 to 5 or 4 led to increased solubility of calcium fluoride on the enamel surface into medium solution. This finding suggests that calcium fluoride on the enamel surface acts as a pH-controlled reservoir of fluoride.  Figure 4(b) shows that in response to the S-PRG coating material, F ions aggregated on the surface of the enamel specimens. S-PRG filler-containing tooth-coating material also functions as a calcium fluoride-like substance on the tooth surface and may release low concentrations of fluoride ions around the coating material surface.

Most restorative treatments, such as resin fillings, lead to the growth of cariogenic bacteria. Saku et al. [17] reported that the adherence of radiolabeled bacteria to the saliva-coated resin surface was significantly lower in S-PRG filler-containing composite resin than in other filling materials. Yoneda et al. [18] also reported that S-PRG eluate suppresses streptococcal adherence and inhibits the protease and coaggregation activities of *Porphyromonas gingivalis*. They suggested that the S-PRG filler-containing material reduces dental plaque formation and bacterial adherence. In the present study, ICP emission spectrometry showed that Sr, B, and Al were present in the demineralized solution. We speculated that these ions inhibit bacterial adhesion on the tooth surface. However, determining the mechanism of this interference will require further investigation.

Despite reports that the frequent use of low concentrations of fluoride agents, such as fluoride-containing toothpaste, is the most beneficial method of preventing demineralization [12], fluoridated dentifrice has failed to fully prevent demineralization [41]. Hausen et al. [44] found no evidence for the effects of fluoride agents on controlling caries in high-risk individuals. The ion-releasing potential of S-PRG filler-containing tooth-coating material may contribute to the prevention of tooth enamel demineralization.

## Figures and Tables

**Table 1:** Mean ion concentrations (ppm) in demineralization solution as determined by ICP emission spectrometry.

	Al	B	Ca	P	Si	Sr	F
Control group	0.23 (±0.05)	0.95 (±0.09)	164.61 (±13.16)	88.20 (±1.74)	1.75 (±0.21)	0.07 (±0.01)	0.00 (±0.00)
Non-S-PRG filler group	0.18 (±0.03)	0.90 (±0.11)	172.53 (±3.53)	90.98 (±1.35)	1.70 (±0.09)	0.08 (±0.01)	0.00 (±0.00)
S-PRG filler group	2.91 (±0.96)	2.29 (±0.48)	162.30 (±0.96)	88.01 (±1.95)	4.38 (±0.98)	9.16 (±3.05)	0.34 (±0.07)
